# Metabolomic Profiling of Tyrosine Kinase Inhibitor-Induced Endothelial Dysfunction and Cardiovascular Toxicity

**DOI:** 10.3390/metabo16030200

**Published:** 2026-03-17

**Authors:** Gurkaranvir Singh, Inderjeet Bharaj, Joey Bettencourt, Amarjit Kaur Sekhon, Gurparvesh Singh, Aaron Sidhu, Emanuel Zayas Diaz, Sulaiman Paika, Ariel De Leon, Ajit Brar, Gursimran Brar, Inderbir Padda, Ambar Andrade

**Affiliations:** 1 Department of Medicine, School of Medicine, Creighton University, Phoenix, AZ 85012, USA; karansingh1@creighton.edu (G.S.); goraya.garry09@gmail.com (G.S.); 2 Department of Medicine, Abrazo Health Network, Glendale, AZ 85308, USA; joey.bettencourt@abrazohealth.com (J.B.); amarjitkbath@gmail.com (A.K.S.); emanuelzayas36@gmail.com (E.Z.D.); smpaika99@gmail.com (S.P.); azdeleon18@gmail.com (A.D.L.); 3 Department of Hematology and Oncology, Cleveland Clinic Weston Hospital, Weston, FL 33331, USA; asidhu23@gmail.com; 4 Department of Medicine, Hurley Medical Center, Michigan State University, Flint, MI 48503, USA; ajitbrar466@gmail.com; 5 Department of Medicine, Punjab Institute of Medical Sciences and Research, Jalandhar 144001, Punjab, India; gursimranbrar009@gmail.com; 6 Department of Internal Medicine, Richmond University Medical Center/Mount Sinai, Staten Island, NY 10310, USA; drpadda91@gmail.com; 7 Department of Cardiology, Banner University Medical Center, University of Arizona, Phoenix, AZ 85004, USA; ambar.andrade@bannerhealth.com

**Keywords:** tyrosine kinase inhibitors, metabolomics, cardiotoxicity, endothelial dysfunction, L-carnitine

## Abstract

Background: Tyrosine kinase inhibitors (TKIs) have transformed cancer therapy; however, they are associated with cardiovascular toxicity. Metabolomics provides a comprehensive framework for identifying early biochemical disruptions that precede clinical manifestations and for formulating mechanism-based intervention strategies. Methods: We conducted a narrative synthesis of published preclinical and translational studies on TKI cardiotoxicity, focusing on untargeted and targeted metabolomic findings and complementary proteomic and transcriptomic data. Functional validation was performed using rodent and cellular models. Mechanistic themes were identified, and implications for biomarker panels, multi-omic integration, and metabolomics-guided interventions were proposed. Conclusions: Metabolomic analyses of various TKIs identified convergent signatures along three interconnected axes: (1) mitochondrial bioenergetic dysfunction characterized by impaired long-chain fatty acid oxidation and adenylate depletion; (2) disruption of endothelial nitric oxide signaling with redox imbalance, including increased nitrotyrosine, Nox activation, and eNOS uncoupling; and (3) an inflammatory metabolic profile marked by elevated branched-chain and aromatic amino acids, creatine, and osmolytes. Rodent models of sunitinib and sorafenib replicate these signatures and demonstrate histological injury, contractile dysfunction, and fibrosis. Preclinical intervention data, particularly restoration of myocardial carnitine, AMPK signaling, and fatty acid oxidation by L-carnitine, provide proof of concept for metabolomics-guided cardioprotection. Metabolomics can identify mechanistic biomarkers that facilitate the early detection, risk stratification, and targeted prevention of TKI-induced cardiovascular injury. Translation into precision cardio-oncology requires prospective validation, standardized assays, and biomarker-driven interventional trials.

## 1. Introduction to Tyrosine Kinase Inhibitors and Cardiovascular Adverse Effects

### 1.1. Background and Mechanism of Action

Tyrosine kinase inhibitors (TKIs) represent a major advancement in targeted cancer therapy. These small-molecule agents competitively inhibit the ATP-binding site of tyrosine kinases, blocking downstream signaling pathways such as MAPK, PI3K–AKT, and JAK–STAT that drive malignant cell proliferation and survival [1,2]. The clinical impact of TKI therapy extends to a wide range of malignancies. Since the introduction of imatinib for chronic myeloid leukemia, more than 50 TKIs have been approved for malignancies including renal cell carcinoma, hepatocellular carcinoma, non-small cell lung cancer, and thyroid cancer [3]. These agents span more than ten distinct pharmacological classes based on their primary kinase targets: BCR-ABL inhibitors (imatinib, dasatinib, nilotinib, bosutinib, ponatinib), VEGFR-targeting multikinase inhibitors (sunitinib, sorafenib, pazopanib, axitinib, cabozantinib, regorafenib, lenvatinib), EGFR/HER2 inhibitors (erlotinib, gefitinib, afatinib, osimertinib, lapatinib, neratinib), ALK/ROS1/MET inhibitors (crizotinib, alectinib, brigatinib, lorlatinib), FGFR inhibitors (erdafitinib, pemigatinib), RET inhibitors (selpercatinib, pralsetinib), BTK inhibitors (ibrutinib, acalabrutinib, zanubrutinib), JAK inhibitors (ruxolitinib, fedratinib), FLT3 inhibitors (midostaurin, gilteritinib), and CDK4/6 inhibitors (palbociclib, ribociclib), though the latter act on serine/threonine rather than tyrosine kinases.

Despite their targeted design, TKIs are not entirely selective [4]. Many function as multikinase inhibitors or share structural similarities that promote off-target binding to kinases expressed in cardiovascular tissues [5]. Inhibition of the vascular endothelial growth factor receptor (VEGFR), platelet-derived growth factor receptor (PDGFR), and related kinases disrupts endothelial homeostasis, angiogenesis, vascular permeability, and cell survival [6]. This leads to endothelial dysfunction, increased vascular stiffness, impaired repair mechanisms, and a spectrum of cardiovascular toxicities including hypertension, thromboembolism, and myocardial ischemia [6].

### 1.2. Clinical Burden of Cardiovascular Toxicity

Cardiovascular (CV) complications are among the most significant non-oncologic adverse effects of TKIs and can limit therapy continuation [7]. Hypertension is the most frequent toxicity, particularly with VEGFR-directed TKIs, such as sunitinib, sorafenib, pazopanib, and axitinib [8]. The incidence of any-grade hypertension exceeds 40% in some trials, with up to 10% developing grade 3–4 hypertension [9]. VEGF inhibition reduces endothelial nitric oxide synthase (eNOS) activity and prostacyclin release, increasing peripheral resistance and vascular stiffness [10].

Clinical trials and observational studies report heart failure and left ventricular systolic dysfunction in approximately 3–8% of patients treated with VEGFR TKIs, with a higher risk after cumulative exposure and in patients with pre-existing cardiovascular risk factors, such as hypertension, coronary artery disease, or prior cardiotoxic therapy [11]. Clinical presentations range from asymptomatic declines in left ventricular ejection fraction detected on surveillance imaging to overt symptomatic cardiomyopathy and heart failure. The onset can be early (weeks) or delayed (months), depending on the agent and patient vulnerability [12]. The proposed mechanisms include mitochondrial dysfunction, impaired fatty acid oxidation (FAO), microvascular rarefaction, and off-target kinase inhibition [11,12,13]. These molecular and metabolic disturbances reduce the myocardial energetic reserve and increase susceptibility to impaired contractility, especially when compounded by hypertension or ischemia [13].

Arterial and venous thrombotic events have been reported with multikinase and VEGFR-targeting TKIs [14]. These events range from isolated venous thromboembolism to severe arterial thrombosis. A meta-analysis by Wu et al. [15] indicated a twofold increased risk of arterial thrombotic events compared to control groups. Furthermore, individual trials and pharmacovigilance studies have reported significant occurrences of myocardial infarction and ischemic stroke in patients treated with these agents, particularly in those with pre-existing cardiovascular risk factors or concurrent prothrombotic conditions [16].

QT prolongation is another class effect, with ~29% of patients experiencing QTc prolongation and ~5% developing life-threatening arrhythmias [17,18]. The most frequently implicated agents include nilotinib, dasatinib, vandetanib, sunitinib, lapatinib, pazopanib, and osimertinib. Nilotinib and vandetanib carry a particularly high risk, with boxed warnings for QT prolongation and sudden cardiac death [19].

A WHO VigiBase analysis identified hypertension, heart failure, and ischemic events as the predominant CV toxicities across > 30 TKIs, with VEGFR inhibitors carrying the highest relative risk [20]. These toxicities may necessitate dose reduction, interruption, or discontinuation of life-prolonging therapy, highlighting the importance of early recognition and prevention.

Beyond cardiovascular toxicity, TKIs are associated with a spectrum of additional adverse drug reactions (ADRs) that complicate clinical management [21]. Hematological toxicities are common across TKI classes: myelosuppression (neutropenia, thrombocytopenia, and anemia) occurs frequently with ABL inhibitors such as imatinib, dasatinib, and nilotinib, reflecting on-target effects on hematopoietic progenitor kinases [22]. Ibrutinib and other BTK inhibitors carry a well-recognized risk of major bleeding and atrial fibrillation due to off-target effects on platelet signaling and cardiac ion channels. Hepatotoxicity, ranging from asymptomatic transaminase elevation to drug-induced liver injury (DILI) and, rarely, acute hepatic failure, has been reported with regorafenib, pazopanib, lapatinib, and imatinib; pazopanib in particular carries a boxed warning for severe and fatal hepatotoxicity [23]. Dermatological reactions (hand-foot skin reaction with sorafenib and sunitinib), hypothyroidism (with sunitinib, sorafenib, and cabozantinib), and gastrointestinal toxicities including diarrhea, mucositis, and nausea are additional class effects that can limit treatment adherence and necessitate dose reductions. Awareness of this broader ADR profile is essential for comprehensive patient monitoring and safe TKI administration.

### 1.3. Pathophysiologic Underpinnings

TKI-related CV injury arises from combined endothelial and myocardial toxicity. VEGF and PDGF inhibition impairs endothelial NO bioavailability, disrupts vascular integrity, and promotes vasoconstriction, hypertension, and a prothrombotic endothelial phenotype [24,25]. Reduced microvascular perfusion and increased afterload amplify myocardial stress [5]. At the cardiomyocyte level, many TKIs induce mitochondrial injury, reactive oxygen species (ROS) generation, endoplasmic reticulum stress, inflammatory signaling, and altered substrate utilization, particularly impaired FAO [24,26,27]. These changes reduce energetic reserve and increase susceptibility to contractile dysfunction. Clinically, this dual vascular-myocardial toxicity explains why some patients develop predominantly hypertension and ischemic events, while others manifest acute declines in left ventricular function or cardiomyopathy [5].

Unlike the largely dose-dependent myocyte necrosis seen with anthracyclines, TKI-associated cardiotoxicity is often at least partially reversible if detected early and the offending agent is interrupted. The risk is higher with cumulative exposure, pre-existing cardiovascular diseases, and concomitant cardiotoxic therapies. Persistent endothelial injury, untreated hypertension, or repeated exposure can produce permanent vascular remodeling and chronic heart disease (Table 1) [10].

### 1.4. Monitoring Challenges

Despite the growing awareness, cardiovascular monitoring during TKI therapy remains inconsistent because of several practical and scientific challenges.

- Baseline risk assessment
  Comorbidities such as hypertension, diabetes, and prior cardiotoxic therapy complicate attribution of cardiac injury to TKIs [34]. Oncology trials often under-represent older adults and those with established cardiovascular disease, limiting trial-derived estimates of TKI cardiotoxicity in real-world, multimorbid populations [35,36]. Risk stratification instruments specifically validated for predicting TKI-related cardiovascular events are lacking; therefore, clinicians must rely on a combination of general cardiovascular risk scores, individual comorbidity profiles, medication review, and treatment-specific toxicity patterns when estimating risk [37].
- Heterogeneity of toxicity across TKI classes
  The cardiovascular risk profile varies widely depending on the target specificity, off-target kinase inhibition, and pharmacokinetics [38]. These differences produce distinct clinical outcomes, such as prominent hypertension and heart failure with VEGFR inhibitors versus arrhythmias, QT prolongation, and vascular occlusive disease with some BCR-ABL inhibitors [39,40]. Therefore, surveillance must be tailored to the agent, dose, and patient comorbidities, rather than applying a single monitoring schedule across all TKIs [41].
- Limitations of conventional monitoring tools
  Current surveillance for TKI cardiotoxicity relies on clinic blood pressure checks, cardiac biomarkers such as troponin and NT-proBNP, ECGs and imaging modalities like echocardiogram with global longitudinal strain and cardiac Magnetic Resonance Imaging [37]. While this strategy is the clinical standard, it often only identifies established or evolving injuries rather than the earliest pathophysiological changes. Subclinical endothelial dysfunction, microvascular impairment, or metabolic derangements within cardiomyocytes frequently precede measurable alterations in left ventricular structure or conventional biomarker elevation, limiting opportunities for truly pre-emptive intervention [42]. This gap is compounded by the lack of validated TKI-specific early biomarkers or imaging end points. Consequently, surveillance strategies rely on pragmatic combinations of clinical assessment, biomarker trends, and modality-specific thresholds, supplemented by expert cardio-oncology input for patients at higher risk [36,43].
- Integration into oncology workflow
  Routine cardiovascular monitoring requires multidisciplinary coordination between oncologists and cardiologists, which is often limited in resource-constrained or outpatient settings [44]. Furthermore, overlapping toxicities from concomitant cancer therapies, such as immune checkpoint inhibitors and anthracyclines, further complicate clinical interpretation by producing similar presentations and obscuring attribution to a single agent, which can delay appropriate management or lead to unnecessary treatment interruptions [43]. Therefore, practical implementation requires pragmatic referral triggers, locally adapted monitoring protocols that prioritize high-risk patients, and streamlined communication channels between teams to ensure timely investigations and risk-based interventions [37].
- Research and guideline gaps
  Existing international guidelines endorse baseline cardiovascular evaluation and periodic monitoring for patients receiving potentially cardiotoxic TKIs; however, the evidence specific to TKIs is limited because prospective, TKI-focused cardio-oncology trials are scarce and often underpowered [34,45]. Furthermore, the definitions and endpoints for “cardiotoxicity” vary widely across studies, making comparisons and meta-analyses difficult. This heterogeneity has impeded the development of validated TKI-specific risk scores, standardized surveillance algorithms, and clear thresholds for treatment interruption. Therefore, uniform outcome definitions and adequately powered prospective studies that include older adults and patients with pre-existing cardiovascular disease are urgently needed.

## 2. Review Methodology

This narrative review was conducted following a systematic literature search of PubMed/MEDLINE, Embase, and Web of Science databases from inception through December 2024. Search terms included combinations of: “tyrosine kinase inhibitor,” “TKI,” “sunitinib,” “sorafenib,” “imatinib,” “metabolomics,” “metabolome,” “cardiotoxicity,” “cardiovascular toxicity,” “endothelial dysfunction,” “carnitine,” “fatty acid oxidation,” and “biomarker.” Additional records were identified through reference lists of retrieved articles and relevant review articles. Studies were included if they reported original metabolomic data (untargeted or targeted) in the context of TKI administration and cardiovascular or metabolic outcomes, or if they provided mechanistic data relevant to TKI-induced cardiotoxicity. Case reports and purely clinical pharmacological studies without metabolomic endpoints were excluded. Two authors independently screened titles and abstracts, with full-text review for potentially eligible studies. Discrepancies were resolved by consensus. Evidence strength for each mechanistic axis was graded informally based on consistency of findings across multiple independent datasets, availability of functional validation data (i.e., genetic or pharmacological perturbation confirming causality), and extent of translational corroboration from human or large animal studies. Findings supported only by single preclinical studies or purely correlative associations are explicitly identified as hypothesis-generating rather than established mechanisms.

## 3. Metabolomic Methodologies in Cardiovascular Research

Metabolomics, a comprehensive study of small molecules produced by metabolic processes, allows for both targeted and untargeted analyses of biological samples. Metabolomics has become an important tool for understanding how the heart and blood vessels respond to diseases. By studying the small molecules involved in metabolism, researchers can identify the biochemical changes that occur early in cardiovascular conditions, such as heart failure, ischemia, and atherosclerosis. These findings can help identify new biomarkers and guide the development of more targeted therapies. Among the various analytical techniques employed in metabolomics, Mass Spectrometry (MS) and Nuclear Magnetic Resonance (NMR) spectroscopy are the most prominent, each with distinct advantages and limitations that influence their application in cardiovascular studies.

### 3.1. Mass Spectrometry

Mass spectrometry (MS) is a widely used analytical technique in metabolomics because of its sensitivity and specificity [46]. When paired with separation methods such as liquid chromatography (LC–MS) or gas chromatography (GC–MS), it can detect a wide range of metabolites, including those present in very low concentrations. It involves three main components: an ion source, mass analyzer, and detector. Ionization techniques such as electrospray ionization (ESI) and matrix-assisted laser desorption/ionization (MALDI) are utilized for different sample states, whereas electron ionization (EI) is typically employed after gas chromatography separation [47].

Mass spectrometry has been instrumental in advancing our understanding of cardiovascular diseases. For instance, studies have identified distinct metabolic signatures associated with conditions such as acute decompensated heart failure and chronic heart failure, leading to the discovery of potential novel biomarkers such as 1-methyl histidine and 3-indolepropionic acid [48,49]. Moreover, lipidomics, a specialized branch of metabolomics that focuses on lipid analysis, leverages mass spectrometry to elucidate disease-associated lipid profiles and the mechanisms underlying cardiovascular disease risks [47].

MS platforms differ importantly in their resolving power and metabolome coverage. High-resolution MS (HRMS) instruments, including Orbitrap (e.g., Orbitrap Fusion, Q Exactive) and quadrupole time-of-flight (Q-TOF) analyzers, provide mass accuracy typically below 5 parts per million, enabling confident molecular formula assignment and discrimination of isobaric metabolites. These platforms are preferred for untargeted discovery metabolomics because they capture the broadest chemical space with minimal a priori knowledge [50]. By contrast, unit-resolution instruments such as triple quadrupole (QqQ) and ion trap analyzers sacrifice mass accuracy for superior sensitivity in targeted assays [50]. Triple-quadrupole multiple-reaction monitoring (MRM) methods on LC-MS/MS platforms are the gold standard for absolute quantification of predefined metabolite panels, offering dynamic ranges of several orders of magnitude and limits of quantification in the low nanomolar range—properties essential for biomarker validation and clinical implementation. In practice, an integrated tiered workflow—initial HRMS discovery followed by QqQ-based targeted validation—maximizes both breadth and rigor in cardiovascular metabolomics research.

### 3.2. Nuclear Magnetic Resonance Spectroscopy

Nuclear Magnetic Resonance (NMR) spectroscopy is a powerful analytical technique that exploits the magnetic properties of atomic nuclei in the presence of a strong magnetic field [51]. NMR spectroscopy relies on the principle that certain nuclei absorb electromagnetic energy at specific frequencies when exposed to a magnetic field. Upon absorption, nuclei transition between spin states, generating resonance frequencies that inform the chemical environment, thus providing detailed chemical insights into molecular structures and dynamics [46]. Compared to MS, it is less sensitive but offers excellent reproducibility. It does not destroy the sample and requires minimal preparation, making it a good option for studies that require consistent and repeatable measurements [52].

NMR is particularly noted for its ability to provide reproducible results across different laboratories, making it a standard method in metabolomic studies. Moreover, NMR offers precise quantification and superior compound identification, enabling researchers to study metabolic pathways in depth [53]. NMR techniques can be adapted for various applications, including targeted metabolomics, which is useful for monitoring specific metabolic changes in longitudinal studies [54].

NMR-based metabolomics encompasses both one-dimensional (1D) and multi-dimensional (2D) experimental approaches, each suited to distinct analytical objectives [55]. One-dimensional 1H-NMR, particularly with Carr-Purcell-Meiboom-Gill (CPMG) pulse sequences to suppress macromolecular background, is the workhorse of high-throughput plasma and urine profiling, yielding semi-quantitative metabolite fingerprints from hundreds of samples per day [55]. Quantitative 1D experiments (noesypr1d, zgpr) enable absolute concentration determination using internal standards or electronic reference methods. 13C-NMR, though inherently less sensitive, provides superior spectral resolution for structural elucidation and is used in conjunction with isotope-labeled substrates for metabolic flux analysis. Two-dimensional experiments substantially increase chemical shift dispersion and resolving power for complex biological matrices: 1H-1H TOCSY and COSY reveal scalar coupling networks for spin-system assignment; 1H-13C HSQC and HMBC experiments correlate proton signals to their one- and multiple-bond carbon partners, enabling unambiguous identification of overlapping resonances common in plasma. Diffusion-ordered spectroscopy (DOSY) further separates metabolites by hydrodynamic radius, aiding the de-convolution of macromolecular and small-molecule signals. When compared with MS-based metabolomics, NMR offers superior structural confirmation and absolute quantification without the need for reference standards, but lower sensitivity (typical detection limits ~1 microM versus nM for LC-MS/MS) and narrower metabolome coverage [56]. A complementary strategy combining NMR for quantitative central metabolites and LC-HRMS for broad lipid and amino acid profiling captures the widest metabolic landscape and is increasingly adopted in multi-platform cardiovascular metabolomics studies [56].

### 3.3. Sample Types: Plasma, Urine, and Myocardial Tissue

The type of biological sample used depends on the biological question, feasibility, and whether systemic or organ-specific information is required.

- Plasma or serum samples are most often used because they are minimally invasive to collect and reflect the overall metabolic state of the body. Changes in plasma amino acids, lipids, and other metabolites are associated with the severity and prognosis of heart failure [57].
- Urine is another useful non-invasive sample type. It reflects both systemic metabolism and renal function. It is particularly valuable for longitudinal monitoring, population screening, and studies in which metabolite clearance or kidney function is relevant [58].
- Myocardial tissue provides the most direct insight into cardiac metabolism, revealing shifts between fatty acid oxidation and glycolysis, accumulation of ischemic intermediates, and remodeling-related metabolic changes [59]. However, human myocardial samples are difficult to obtain, limiting most tissue-level insights to animal models, explanted hearts, or small biopsy series [60]. Therefore, tissue studies are often complementary to plasma/urine work and are critical for the mechanistic validation of circulating biomarkers.

Practical considerations include invasiveness versus organ specificity, temporal resolution for serial sampling, and the need to integrate circulating and tissue data to distinguish cardiac-specific signals from systemic or multi-organ influences [59]. Hence, combining multiple sample types when possible provides the clearest metabolomic view of cardiovascular disease. Therefore, the study design should align sample selection with analytical platforms, statistical power, and validation strategies across cohorts.

Cross-platform variability and pre-analytical factors represent critical methodological challenges that affect the reproducibility and comparability of metabolomic findings across studies. Platform-specific differences in metabolome coverage, ionization efficiency, and chromatographic selectivity mean that metabolite panels identified on one instrument class (e.g., Orbitrap-based HRMS) may not be directly transferable to another (e.g., Q-TOF or triple quadrupole). Batch-to-batch analytical drift, variable sample storage conditions, freeze–thaw cycling, and differences in sample preparation protocols (protein precipitation versus solid-phase extraction versus protein binding) introduce systematic biases that can inflate or suppress apparent metabolite differences. Quality control samples (pooled biological QC injected throughout each analytical batch), internal standards, and batch-correction algorithms (e.g., LOESS regression, ComBat) are essential for reliable cross-study comparisons. Metabolite identification confidence should be reported according to the Metabolomics Standards Initiative (MSI) classification [61,62]: level 1 (confirmed by authentic standard), level 2 (putatively annotated by spectral library match), or level 3 (putatively characterized by compound class). In the current review, many cited studies employ HRMS untargeted platforms with MSI level 2 identification, and readers should interpret reported metabolite signatures with awareness of these limitations when comparing results across independent datasets.

## 4. Preclinical Metabolomic Insights into TKI Toxicity

### 4.1. Key Metabolic Pathways Disrupted by TKIs

TKIs have transformed oncology but carry a persistent, low-frequency risk of cardiotoxicity. Recent advances in metabolomics offer a novel approach for analyzing such perturbations. Preclinical metabolomic studies in rodents have begun to map the biochemical landscape of this adverse effect, revealing consistent perturbations across three interrelated metabolic domains: cellular energy metabolism, lipid handling and membrane composition, and amino acid–dependent antioxidant and signaling pathways [63].

- Mitochondrial Energy Metabolism
  A hallmark finding across multiple TKI exposures is the depletion of adenylate and purine intermediates (e.g., AMP, adenosine, inosine, and adenine), consistent with the depletion of the cellular adenylate pool and impaired oxidative ATP generation [63,64]. Concomitant ultrastructural abnormalities, including mitochondrial swelling, cristae disruption, and sarcomeric disorganization, result in direct mitochondrial damage and reduced bioenergetic reserve [65]. These changes may contribute to early declines in contractility, even in the absence of significant cell death in cardiomyocytes.
- Lipid metabolism and membrane integrity
  Sunitinib and other VEGFR/PDGFR inhibitors are associated with significant reductions in myocardial long-chain polyunsaturated fatty acids (PUFAs), such as DHA and EPA, as well as downstream phospholipid intermediates [63]. The loss of PUFAs and specific phospholipids impairs membrane fluidity, mitochondrial membrane function, and anti-inflammatory signaling, thereby lowering cellular resilience to metabolic and oxidative insults. Sorafenib has also been linked to changes in lipid-related metabolites including stearamide, indicating broader perturbations in fatty acid oxidation and membrane remodeling [66].
- Amino acid metabolism and redox homeostasis
  Amino acid metabolism, particularly involving sulfur-containing and antioxidant-related species, is frequently disrupted [63,64]. Taurine depletion has been observed in both cardiac and skeletal muscles in rodent models of TKI exposure, particularly with sorafenib and imatinib, which undermine calcium handling, osmoregulation, and antioxidant buffering [67]. Perturbations in methionine, homocysteine, and cysteine levels indicate interference with the transsulfuration pathway and glutathione biosynthesis, amplifying oxidative stress susceptibility [67]. Collectively, these amino acid alterations diminish endogenous defenses against reactive oxygen species and impair excitation–contraction coupling.

The intersection of mitochondrial dysfunction, lipid depletion, and impaired amino-acid-based antioxidant systems yields a coherent pathophysiological model: TKIs induce energetic insufficiency and membrane instability while weakening redox defenses, thereby lowering myocardial tolerance to physiological and ischemic stress. These metabolic fingerprints are detectable across multiple TKI classes and experimental contexts, making them promising candidates for early biomarker development and testing of targeted mitigation strategies (e.g., PUFA or taurine supplementation and mitochondrial protective agents).

### 4.2. Rodent Models of Sunitinib and Sorafenib Cardiotoxicity: Phenotypes, Mechanisms, and Experimental Considerations

Rodent models have been instrumental in delineating the multifactorial nature of sunitinib and sorafenib-associated cardiotoxicity, revealing context-dependent phenotypes that range from subclinical metabolic derangement to myocyte necrosis and heart failure. These models combine in vivo functional assessment, histology, and metabolomics to link biochemical changes to structural and functional outcomes.

- Sorafenib models
  In rodent preclinical studies, sorafenib has been shown to be implicated in the development of cardiotoxicity, both indirectly and directly, especially when combined with additional cardiac stresses, such as MI. Duran et al. [68] treated mice with sorafenib for three weeks, induced myocardial infarction after one week of treatment, and observed a sharp increase in two-week mortality despite the rodents having pre-treatment preserved echocardiographic ejection fraction. Histologically, sorafenib-treated hearts had reduced heart weights and chamber volumes but increased cardiomyocyte cross-sectional areas, compatible with an overall loss of myocytes with compensatory hypertrophy of survivors, likely leading to systolic/diastolic dysfunction [68]. Furthermore, in vitro exposure of adult ventricular myocytes to high-dose sorafenib induced necrosis, whereas lower doses did not impair contractile function. This points towards early toxicity resulting from myocyte loss and subsequent compensatory hypertrophy as a method of repair rather than intrinsic systolic impairment. Consequently, echocardiographic evidence of systolic impairment was absent [68].
  In contrast, Jensen et al. [63] treated female FVB/N mice with sorafenib at a similar dose (30 mg/kg/day) for two weeks and observed a modest but statistically significant decline in systolic function, with fractional shortening (FS) decreasing from baseline (~56%) to ~50% by the end of treatment. This decline occurred in the absence of myocardial infarction or ischemic stress and was accompanied by pronounced alterations in cardiac and skeletal muscle metabolism, particularly within the taurine/hypotaurine pathway, as revealed by global metabolomic profiling. These metabolic shifts may contribute directly to impaired myocardial energetics and contractility [63]. Mechanistic exploration in rodent and cellular systems implicates impaired calcium handling, reduced phospholamban Ser16 phosphorylation, diminished sarcoplasmic reticulum calcium content, and activation of endoplasmic reticulum stress pathways (PERK–eIF2α–ATF4), thus resulting in downregulation of mitochondrial complex I subunits, promoting ROS and apoptosis [65,69]. These studies support a multifactorial model in which necrosis, defective repair, metabolic derangement, calcium dysregulation, and endoplasmic reticulum/mitochondrial stress collectively mediate sorafenib cardiotoxicity.
- Sunitinib models
  Sunitinib has been shown to induce cardiotoxicity through a combination of metabolic, mitochondrial, and microvascular mechanisms in several preclinical rodent studies. Jensen et al. [29] treated female FVB/N mice with sunitinib dosed at 40 mg/kg, daily, for two weeks and performed non-targeted metabolomic profiling of cardiac, skeletal muscle, liver, and serum tissues. Analysis revealed a significant depletion of long-chain omega-3 and omega-6 fatty acids, specifically docosahexaenoic acid (DHA) and arachidonic acid/eicosapentaenoic acid (AA/EPA), as well as O-phosphocholine and 6-hydroxynicotinic acid in cardiac tissue compared to controls. Similarly to their study with sorafenib, these metabolic alterations were associated with early diastolic dysfunction observed on echocardiography as a significant reduction in fractional shortening, supporting a link between sunitinib-induced metabolic shifts and contractile impairment [29,63].
  Mechanistic studies have indicated that sunitinib disrupts mitochondrial oxidative phosphorylation and promotes a shift toward glycolysis. This remodeling is associated with increased myocardial FDG uptake and decreased mitochondrial protein expression, effects mediated in part by endothelin-1 signaling and reversible with endothelin receptor antagonism [70]. In vitro studies have implicated CaMKII activation, mitochondrial superoxide generation, and apoptosis in non-myocyte populations, linking metabolic stress to fibrotic remodeling [67].

Although these models provide critical mechanistic insights, they have limitations. Most rodent studies employ short treatment windows and non-tumor-bearing hosts, which may underestimate cumulative toxicity and the influence of cancer-related cachexia or additional systemic inflammation. Nonetheless, the consistent identification of key metabolic derangements, such as omega-3 fatty acid depletion and mitochondrial compromise, suggests candidate biomarkers for early detection and targets for cardioprotection. Future preclinical studies should aim to incorporate tumor-bearing hosts, longer treatment timelines, and test mitigation strategies, such as endothelin receptor antagonism or omega-3 supplementation, to better mirror clinical contexts.

## 5. Mechanistic Pathway Analysis

The cardiotoxic effects of tyrosine kinase inhibitors (TKIs) are multifaceted and primarily result from disruptionions of several key cellular pathways. These pathways contribute to mitochondrial dysfunction, altered calcium homeostasis, and direct cardiomyocyte damage, leading to adverse cardiac events during TKI therapy (Table 2). 

### 5.1. Mitochondrial Bioenergetics and Fatty-Acid Oxidation

Mitochondria are central to cardiomyocyte energy homeostasis, and their disruption is a recurring theme in TKI-induced cardiotoxicity. Preclinical and translational studies have demonstrated that TKIs impair oxidative phosphorylation, reduce ATP generation, and alter substrate preference [73]. In rodent models, exposure to sorafenib and sunitinib has been associated with depletion of adenylate metabolites (AMP, adenosine, and inosine), reflecting contraction of the adenylate pool and impaired mitochondrial ATP synthesis, contributing to compromised cardiac contractility and increased susceptibility to heart failure [30,74]. Additionally, TKIs induce mitochondrial oxidative/nitrosative stress through excessive production of reactive oxygen species (ROS) and reactive nitrogen species [75]. This oxidative stress can trigger mitochondrial fragmentation and loss of membrane potential, further impairing ATP synthesis and exacerbating cardiomyocyte injury [76].

Mitochondrial biogenesis and metabolic adaptations play significant roles in the pathophysiology of TKI-associated cardiomyopathy. Peroxisome proliferator-activated receptor gamma coactivator 1-alpha (PGC-1α) is a vital regulator of mitochondrial biogenesis, and dysregulation of this pathway has been observed following TKI administration. In a study, imatinib treatment decreased PGC-1α levels, disrupting mitochondrial mass and function and leading to compromised cardiac performance [77]. In addition, PGC-1α enhances fatty acid oxidation, which is crucial for energy production in cardiomyocytes. TKIs may inhibit mitochondrial fatty acid synthesis pathways, altering acyl-carnitine flux and β-oxidation capacity [78]. This remodeling forces a metabolic shift toward glycolysis, which is evident in the increased myocardial FDG uptake in sunitinib-treated mice65. Although this shift offers temporary support for energy production, it may have harmful downstream consequences if sustained [30].

### 5.2. Endothelial Nitric Oxide Signaling and Oxidative Stress

Endothelial dysfunction and oxidative stress represent critical pathways in TKI-associated cardiotoxicity. TKIs targeting VEGFR and PDGFR disrupt vascular signaling, impair nitric oxide (NO) bioavailability, and promote reactive oxygen species (ROS) generation [31]. Under physiological conditions, VEGF binding to VEGFR2 activates tyrosine kinase cascades (PI3K → Akt and Ras → ERK signaling), which sustain endothelial survival, angiogenic capacity, and eNOS phosphorylation (Ser^1177) to maintain NO bioavailability and vasodilator tone [79]. VEGFR blockade therefore causes an “on-target” loss of PI3K–Akt–eNOS signaling, reducing eNOS phosphorylation and NO production, and immediately impairing endothelium-dependent vasodilation and antithrombotic function [80]. Concomitantly, VEGFR inhibition shifts the vascular redox balance toward pro-oxidant pathways, and NADPH oxidases (Nox isoforms) and dysfunctional mitochondria become dominant sources of superoxide (O_2_^−^), increasing hydrogen peroxide (H_2_O_2_) and peroxynitrite (ONOO^−^) formation [81]. Superoxide reacts with NO, chemically depleting NO and forming ONOO^−^, which oxidizes lipids, proteins, and cofactors, such as tetrahydrobiopterin (BH4). BH4 oxidation promotes eNOS “uncoupling,” converting eNOS from an NO producer into a net source of O_2_^−^ and establishing a feed-forward loop of oxidative injury [32].

**Figure 1:**
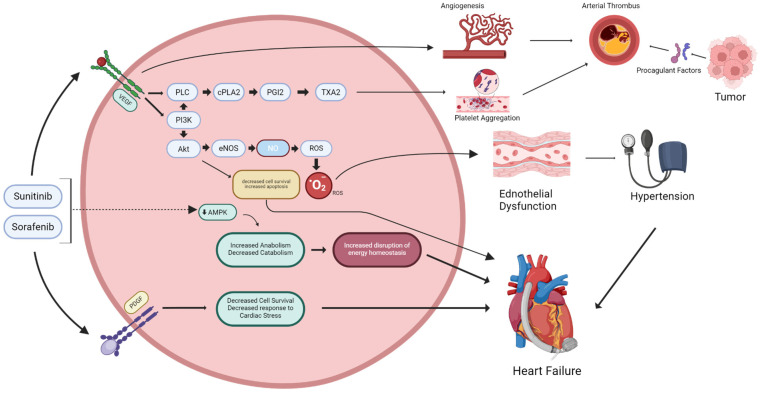
Mechanisms linking TKIs to endothelial dysfunction and myocardial injury. Mechanistic schematic illustrating the role of vascular endothelial growth factor (VEGF) and platelet-derived growth factor (PDGF) receptor signaling in maintaining vascular and myocardial homeostasis, and the pathophysiologic consequences of inhibition by multikinase TKIs such as sunitinib and sorafenib. The diagram integrates endothelial, vascular smooth muscle, and cardiomyocyte signaling nodes to depict how TKI-mediated disruption leads to endothelial dysfunction, oxidative stress, metabolic derangement, and adverse cardiovascular outcomes. Arrows: Solid black arrows represent direct activation or sequential signaling flow; dashed arrows indicate indirect or downstream effects of TKI inhibition; grey arrows link intracellular pathway disruption to tissue- and organ-level clinical sequelae. Colors: The pink oval represents the intracellular signaling compartment. Green labels and boxes denote physiological signaling molecules and protective/homeostatic pathways (e.g., VEGF, NO, PDGF, AMPK). Dark red/maroon boxes denote deleterious or pathological outcomes (e.g., ROS generation, disruption of energy homeostasis, decreased cell survival). The red sphere marked O_2_ represents superoxide/reactive oxygen species. Key clinical sequelae shown at the tissue and organ level include angiogenesis impairment, thrombosis, vascular smooth muscle dysfunction, hypertension, immune-mediated inflammation, and heart failure, contextualizing the metabolomic and functional findings reported in this review.

The loss of NO and increased ROS levels drive endothelial apoptosis and capillary rarefaction, raising peripheral resistance and reducing microvascular perfusion. In the kidney, VEGFR inhibition disrupts podocyte–endothelial crosstalk, causing the loss of glomerular fenestrations and proteinuria. In the heart, microvascular regression reduces coronary reserve and, together with afterload elevation from hypertension, creates ischemic stress that exacerbates myocyte vulnerability [31,32,80]. VEGFR blockade also downregulates Nrf2-mediated antioxidant defenses, further impairing redox buffering and perpetuating inflammation [82]. Finally, endothelial injury fosters prothrombotic and vasospastic states via increased endothelin-1 expression, platelet activation, and upregulation of adhesion molecules, linking molecular redox derangements to clinical sequelae [83]. Consequently, clinical and preclinical data consistently report hypertension, microvascular rarefaction, and impaired vasodilatory responses in patients receiving VEGFR inhibitors (Figure 1) [31,84].

### 5.3. Inflammatory Metabolite Signatures

Elevated circulating amino acids, creatine, and mannitol levels observed in patients receiving TKIs constitute a coherent inflammatory metabolic signature that links systemic metabolic stress to endothelial dysfunction and vascular injury. Increased branched-chain and aromatic amino acids augment mitochondrial substrate flux and can overload the electron transport chain, promoting excessive ROS generation [85]. In the setting of TKI-induced mitochondrial impairment, this ROS surge activates NADPH oxidases (Nox), amplifying superoxide (O_2_^−^) and peroxynitrite (ONOO^−^) formation, and chemically depleting NO [85]. Peroxynitrite-mediated oxidation of tetrahydrobiopterin (BH_4_) drives eNOS uncoupling, converting eNOS into a net source of superoxide rather than NO, thereby collapsing endothelial vasodilatory capacity [31].

Similarly, elevated plasma creatine levels disrupt energy homeostasis and impair oxidative phosphorylation. Creatine accumulation correlates with reduced ATP generation and impaired L-arginine availability, further limiting NO synthesis and exacerbating redox imbalance [31,86]. The combined effects of amino acid-driven mitochondrial overload and creatine-linked energetic failure potentiate the activation of redox-sensitive transcription factors, such as NF-κB, which upregulates vascular adhesion molecules (VCAM-1, ICAM-1), chemokines, and pro-inflammatory cytokines, thus promoting endothelial activation, leukocyte recruitment, barrier dysfunction, and microvascular regression (capillary rarefaction), all hallmarks of TKI-associated vascular injury [85,86].

Elevated plasma mannitol levels indicate osmotic stress and altered endothelial permeability. Hyperosmotic conditions disrupt tight junctions and cytoskeletal organization, thereby increasing vascular leakiness and barrier dysfunction. Mannitol-induced osmotic stress also activates mechanosensitive signaling cascades, including the MAPK and TonEBP/NFAT5 pathways, amplifying pro-inflammatory gene expression and endothelial activation [71,72]. In combination, mannitol-mediated barrier disruption and creatine-mediated mitochondrial oxidative stress synergistically activate inflammatory signaling to promote vascular stiffening, hypertension, and proatherogenic remodeling in patients undergoing TKI therapy.

It is important to explicitly distinguish between well-validated mechanisms and associative metabolite observations throughout this review. Mechanistic axes with direct functional validation that is supported by genetic knockdown, pharmacological perturbation, or isotope tracer confirmation include the AMPK-ACC2-malonyl-CoA-CPT1 axis in sunitinib-exposed hearts (validated through carnitine supplementation rescue experiments) and VEGFR-PI3K-Akt-eNOS uncoupling (validated through pharmacological eNOS inhibition and BH4 supplementation studies). In contrast, the reported elevations in circulating branched-chain amino acids (BCAAs), aromatic amino acids, creatine, and osmolytes represent associative metabolite shifts that are mechanistically plausible but not yet causally established. Similarly, taurine depletion correlates with contractile dysfunction but causal evidence from taurine supplementation or targeted depletion studies in TKI models remains limited. Key experiments needed to advance causal understanding include: (1) endothelial cell-specific genetic manipulation of fatty acid oxidation (e.g., endothelial CPT1a knockout) to assess whether endothelial metabolic flexibility is required for TKI tolerance; (2) direct measurement of NADPH oxidase activity (lucigenin chemiluminescence or electron paramagnetic resonance) and eNOS coupling status (monomeric versus dimeric eNOS by low-temperature SDS-PAGE) in TKI-treated endothelial cells and murine hearts; (3) stable isotope tracing using [U-13C]palmitate, [1,2-13C2]glucose, and [U-13C]leucine in TKI-treated cardiomyocytes and endothelial cells to quantify flux through FAO, glycolysis, and BCAA oxidation and confirm pathway-level impairment rather than pool-size changes alone; and (4) longitudinal multi-time-point metabolomics in tumor-bearing animals to disentangle TKI-specific effects from cancer cachexia and systemic inflammation.

## 6. Translational Perspectives and Future Directions

Integrating metabolomics with proteomics/transcriptomics, developing early warning metabolite panels, and testing metabolomics-guided interventions (notably L-carnitine for sunitinib) are complementary translational paths to reduce TKI cardiotoxicity and enable precision cardio-oncology.

### 6.1. Integration with Proteomics and Transcriptomics

Combining metabolomics with proteomic and transcriptomic datasets strengthens mechanistic inference and biomarker discovery by linking small-molecule changes to upstream regulatory networks and effector proteins [60]. Multi-omic integration can determine whether a metabolite shift reflects altered enzyme abundance, post-translational modification, or transcriptional reprogramming [63]. For example, decreased long-chain fatty acids paired with reduced CPT1 protein or lower CPT1 mRNA levels would indicate impaired LCFA transport rather than increased peripheral clearance [87]. Integrative pipelines should use timed sampling (baseline, early-on-treatment, and longitudinal follow-up) and combination samples (plasma plus tissue when feasible) to map the trajectories from kinase inhibition to metabolic phenotype [29]. Importantly, proteomic and transcriptomic layers help interpret nonspecific metabolite biomarkers (e.g., branched-chain amino acids) by revealing tissue sources and regulatory drivers, thereby improving specificity for cardiac versus systemic effects. Moreover, proteomic analyses have shown that specific proteins, such as cardiac troponin T2, serve as biomarkers for cardiac damage induced by TKIs [88].

The integration of transcriptomics with mechanistic mathematical modeling presents a novel approach to understanding the cardiotoxic effects associated with TKIs. Recent studies have demonstrated that by utilizing a 3′-Digital Gene Expression (DGE) method to prepare mRNA sequencing (mRNAseq) libraries, researchers can effectively quantify gene expression changes in response to various TKIs administered to induced pluripotent stem cell-derived cardiomyocytes (iPSC-CMs) from healthy donors [89,90]. By correlating these computational predictions with experimental data, researchers have confirmed the robustness of this integrated approach, achieving a strong predictive accuracy in metrics related to TKI-induced cardiotoxicity (R^2^ = 0.87) [89]. Additional transcriptomic analyses have revealed altered expression of ion channel genes (e.g., KCNH2/hERG, SCN5A, and CACNA1C), calcium-handling proteins, and extracellular matrix remodeling genes in response to TKIs, which contribute to arrhythmia susceptibility and structural cardiac injury [91,92,93]. The integration of these gene signatures with clinical risk scores and adverse event data could potentially demonstrate their predictive value for TKI-induced cardiotoxicity and generate experimentally testable predictors [94].

### 6.2. Early Warning Panels for Risk Stratification

The establishment of comprehensive risk stratification protocols is essential in the context of TKI therapy, particularly for patients with pre-existing cardiovascular conditions or those receiving concurrent treatment. Early warning metabolite panels aim to identify patients at an elevated risk of TKI cardiotoxicity before irreversible injury occurs. The panels should combine metabolites reflecting complementary axes: mitochondrial energetics (adenylate/purine metabolites, creatine), lipid integrity (DHA/EPA, acyl-carnitines), and redox/inflammatory status (taurine, kynurenine, nitrotyrosine proxies) [85,86,95]. Panel development requires (1) discovery in well-phenotyped preclinical and clinical cohorts, (2) analytic validation on targeted platforms (LC-MS/MS), and (3) prospective clinical validation with predefined thresholds and decision rules. Risk models should integrate clinical covariates (age, baseline CV disease, concomitant cardiotoxic agents), imaging (strain echocardiography), and circulating biomarkers (troponin and NT-proBNP) to improve the positive predictive value.

Candidate biomarker selection should follow explicit criteria: metabolites must demonstrate consistent directional change across at least two independent datasets, biologically plausible linkage to TKI mechanisms, and technical feasibility on validated clinical-grade platforms. Proposed candidate panels include: (i) a bioenergetic panel comprising plasma free carnitine, acetylcarnitine, C18-acylcarnitine, DHA, EPA, and AMP/adenosine; (ii) a redox/inflammatory panel comprising nitrotyrosine, kynurenine/tryptophan ratio, and high-sensitivity CRP; and (iii) an osmotic/vascular panel comprising taurine and, where available, asymmetric dimethylarginine (ADMA) as an eNOS inhibitor [96]. Timing of biomarker assessment should align with pharmacokinetic exposure: baseline (pre-treatment), early on-treatment (2–4 weeks after initiation, when sub-clinical metabolic shifts begin), and at standard clinical monitoring time points (3 and 6 months). Pre-analytical standardization is essential [96]: plasma samples should be collected in EDTA tubes, processed within 30 min of venipuncture, aliquoted in volumes sufficient for duplicate analysis, and stored at −80 °C; urine samples should be spot or first-morning void, volume-corrected to creatinine. A proposed biomarker-guided interventional trial framework would enroll patients initiating VEGFR-TKIs (e.g., sunitinib or sorafenib) and randomize those who develop bioenergetic panel abnormalities at 2–4 weeks to metabolomics-guided intervention (e.g., L-carnitine supplementation) versus standard monitoring, with primary endpoints of serial change in GLS and troponin, and secondary endpoints of metabolite panel normalization, treatment discontinuation rates, and major adverse cardiovascular events.

### 6.3. Metabolomics-Guided Interventions

**Figure 2:**
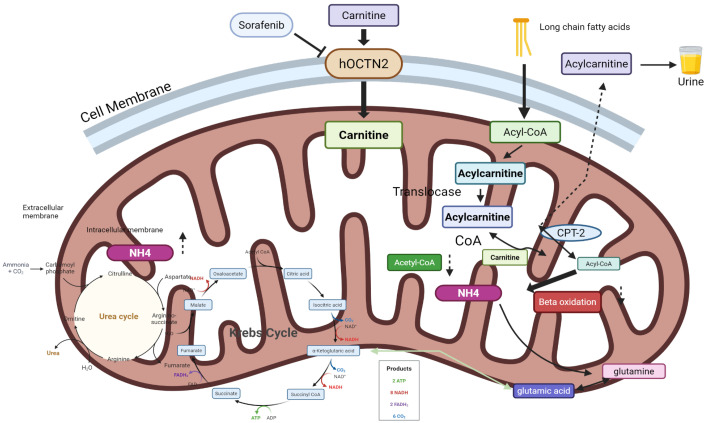
Carnitine-mediated long-chain fatty-acid transport and mitochondrial metabolism: interactions with sorafenib. Schematic representation of the carnitine shuttle and mitochondrial metabolic pathways, illustrating how sorafenib-mediated inhibition of carnitine uptake and mitochondrial function disrupts long-chain fatty acid (LCFA) oxidation, ATP production, and nitrogen handling. The diagram depicts carnitine transport via the human organic cation/carnitine transporter 2 (hOCTN2), conversion of LCFAs to acylcarnitine by carnitine palmitoyltransferase 1 (CPT-1), translocation across the inner mitochondrial membrane, reconversion to acyl-CoA by CPT-2, and subsequent β-oxidation yielding acetyl-CoA for the tricarboxylic acid (TCA/Krebs) cycle and respiratory chain. Sorafenib inhibits hOCTN2 (indicated by the blunted arrow), impairing carnitine uptake and mitochondrial integrity, resulting in altered acylcarnitine profiles, reduced fatty acid oxidation capacity, diminished energy output, and secondary disturbances in glutamine/glutamate metabolism and urea cycle function. Arrows: Solid black arrows indicate direct metabolic or transport steps; the dark grey blunted arrow from sorafenib to hOCTN2 denotes pharmacological inhibition; dashed arrows represent impaired or attenuated pathways resulting from sorafenib exposure; coloured curved arrows within the Krebs cycle denote sequential enzymatic reactions and cofactor production (red arrows for NADH generation, blue arrows for FADH_2_, and green arrows for CO_2_ release). The green arrow from α-ketoglutaric acid to glutamic acid represents the transamination/reductive amination pathway. Colors: Green boxes denote substrates and metabolic intermediates of the carnitine shuttle and Krebs cycle (e.g., carnitine, acylcarnitine, acetyl-CoA, citric acid, oxaloacetate). Blue boxes denote acyl-CoA and acylcarnitine species involved in mitochondrial membrane translocation. Pink/magenta boxes highlight nitrogen-containing metabolites and pathways (NH4, glutamic acid, glutamine, urea cycle). The dark red box denotes β-oxidation. Yellow boxes denote Krebs cycle intermediates. The product box summarises net Krebs cycle output per turn (2 ATP, 8 NADH, 2 FADH_2_, 6 CO_2_). The urine sample icon indicates urinary excretion of acylcarnitine species as a biomarker readout. Abbreviations: hOCTN2, human organic cation/carnitine transporter 2; CPT-1/2, carnitine palmitoyltransferases 1 and 2; CoA, coenzyme A; TCA, tricarboxylic acid cycle; NADH, nicotinamide adenine dinucleotide; FADH_2_, flavin adenine dinucleotide.

Metabolomic analysis has identified several promising metabolites as potential therapeutic targets for TKI-induced cardiotoxicity. Preclinical evidence supports the use of L-carnitine as a metabolomics-guided intervention for sunitinib-induced cardiotoxicity [94]. Sunitinib reduces myocardial carnitine, inhibits AMPK-α2, increases ACC2 activity and malonyl-CoA levels, and thereby suppresses CPT1-mediated LCFA mitochondrial uptake and β-oxidation. These changes coincide with hypertrophy, fibrosis, and elevated cardiac enzymes [94]. In rodent models, oral L-carnitine (200–400 mg/kg/day) restored myocardial carnitine, reactivated AMPK signaling, lowered ACC2 and malonyl-CoA levels, increased CPT1 expression, normalized cardiac enzymes and indices, and reversed fibrosis and inflammatory markers (NOX2, IL-1β, IL-6, TGF-β1) while increasing IL-10 and MMP-9 [94]. These mechanistic and phenotypic reversals support a causal role for carnitine deficiency in sunitinib-induced cardiotoxicity and justify early phase clinical testing. Translational steps include dose-optimizing and safety studies in patients on sunitinib, assessment of pharmacokinetic interactions (e.g., with metformin), and stratification by baseline carnitine status or metabolomic risk scores (Figure 2).

Beyond carnitine, metabolomics can help identify other targeted supplements (PUFAs, taurine, and BH_4_ precursors) or repurposed drugs (AMPK activators and Nox inhibitors) to restore metabolic resilience. However, the application of metabolomics in clinical practice faces challenges, including variability in methodologies and data interpretation, which must be addressed to fully realize its potential in improving patient outcomes [97]. Randomized, biomarker-driven trials that use metabolite panels as entry criteria and mechanistic endpoints (metabolome, imaging, troponin) will be critical to demonstrate clinical benefits and implement metabolomics-guided cardioprotection in oncology practice [29,97]. As the field of metabolomics continues to evolve, its contributions to understanding and managing TKI-induced cardiotoxicity may revolutionize patient-specific treatment strategies in oncology, offering a pathway to improve cancer care and safeguard cardiovascular health in survivors [22].

## 7. Targeted Preventative Treatment Strategies

Targeted preventive treatment for TKI-induced cardiomyopathy prioritizes proactive risk reduction, early detection, and mechanism-directed interventions to preserve both treatment efficacy and cardiovascular health. The integration of baseline risk stratification, longitudinal monitoring, and translational biomarkers enables personalized prevention pathways. These can range from optimized medical therapy and lifestyle modifications to metabolomics-guided nutrition or pharmacological interventions delivered within a multidisciplinary cardio-oncology framework.

### 7.1. Risk Stratification Models and Clinical Integration

Risk stratification should combine conventional cardiovascular scores with cardio-oncology-specific tools to identify patients who require intensified monitoring or prophylaxis. Traditional interventions, such as the Framingham Risk Score and coronary artery calcium scoring, remain useful for baseline atherosclerotic risk classification and inform modifiable risk optimization prior to TKI initiation [37]. However, dedicated cardio-oncology tools outperform general population scores in predicting treatment-related cardiotoxicity. The HFA/ICOS risk assessment integrates comorbidities, biomarkers, ECG, and imaging to produce individualized risk categories and has demonstrated superior sensitivity in TKI-treated cohorts [34]. Furthermore, emerging clinicogenomic models add discriminative power by incorporating genetic variants linked to arrhythmia or contractile vulnerability (e.g., ion channel and cardiomyopathy-associated loci) [98]. When combined with clinical variables (age, hypertension, prior cardiotoxic therapy), these models can refine pre-treatment risk estimates and guide prophylactic choices, such as the earlier initiation of cardioprotective drugs or enrollment in intensified surveillance pathways [98]. A multidisciplinary approach (oncology, cardiology, and genetics) is essential to interpret genomic findings and balance oncologic benefits against cardiovascular risk [34,43].

### 7.2. Monitoring Strategy and Multidisciplinary Management

Structured longitudinal monitoring is critical for the early detection and appropriate intervention of TKI-induced cardiotoxicity. For patients stratified as intermediate- or high-risk, a more rigorous approach would be beneficial [37]. One approach could be implementing scheduled surveillance with ECG, biomarker panels (high-sensitivity troponin, NT-proBNP), and echocardiography, including strain imaging at baseline, 3, 6, 12 months, and then annually or as clinically indicated [36,43]. The frequency should be individualized according to the risk tier and agent potency. If the biomarkers rise or there is a decline in strain or cardiac function on TTE, it should trigger expedited evaluation and consideration of therapy modification or cardioprotective initiation [98].

Furthermore, a multidisciplinary care pathway that embeds cardio-oncology within oncology clinics would be an appropriate approach. This would facilitate rapid decision-making regarding dose adjustments, temporary treatment holds, or initiation of ACE inhibitors, ARBs, or β-blockers for cardioprotection. Consensus statements recommend prophylactic cardioprotective therapy for selected high-risk patients and endorse shared decision-making that weighs cancer control against cardiovascular safety [34,98]. Implementation requires clear referral criteria, rapid access to imaging and biomarkers, and documentation of monitoring plans in the oncology treatment pathway to be effective.

### 7.3. Predictive Translational Models and Implementation

Translational predictive platforms accelerate the risk prediction and stratification of novel TKIs by combining human-relevant in vitro systems, quantitative modeling, and multiparametric readouts. Effective implementation requires three interlocking components: biologically relevant assay systems, integrative computational models, and rigorous clinical validation. hiPSC-CMs provide a scalable and innovative model for studying cardiotoxicity, as these cells mirror the human cardiac environment more accurately than traditional animal models [89]. By utilizing hiPSC-CMs, researchers have elucidated the cardiotoxic mechanisms of different TKIs and correlated these findings with proteomic data [99]. Complementary assays, including high-content imaging, extracellular flux analysis (Seahorse), and targeted metabolomics, capture mitochondrial respiration, substrate utilization, and redox responses that are central to TKI toxicity [73,94].

In vitro to in vivo extrapolation (IVIVE) frameworks combine hiPSC-derived cardiomyocyte assays with quantitative systems pharmacology (QSP). By simulating tissue exposures, off-target kinase engagement, and downstream pathway perturbations (e.g., AMPK-ACC2-CPT1 axis), QSP frameworks can predict the likelihood and time course of functional impairment under clinically relevant dosing regimens [100]. In addition, multiparametric high-throughput panels (contractility, calcium handling, mitochondrial readouts, and apoptosis) integrated with clinical and genomic data can be used to train machine-learning risk models that predict individual susceptibility and guide personalized monitoring intensity [91].

Translational models must progress through analytic validation (assay precision and reproducibility), clinical validation (prospective cohorts demonstrating predictive performance), and demonstration of clinical utility (improved outcomes or cost-effectiveness). Prospective biomarker-driven pilot studies that embed metabolomic and functional endpoints alongside imaging and troponin/NT-proBNP are critical. For clinical implementation, predictive outputs should be integrated into electronic health records with clear action thresholds and care pathways, enabling automated alerts, standardized monitoring schedules, and rapid referral to cardio-oncology services.

## 8. Conclusions

This review synthesizes preclinical and translational metabolomic evidence that tyrosine kinase inhibitors (TKIs) induce a convergent set of metabolic and vascular perturbations that underlie cardiovascular toxicity. Three underlying interrelated mechanisms, namely, mitochondrial bioenergetic failure with impaired long-chain fatty-acid oxidation, endothelial nitric oxide signaling disruption with redox imbalance and microvascular rarefaction, and an inflammatory-metabolic signature characterized by amino-acid, creatine, and osmolyte dysregulation, emerge consistently across rodent, cellular, and human datasets. These biochemical alterations are associated with histologic injury, contractile dysfunction, and adverse remodeling and provide mechanistic targets for intervention.

Translational integration of metabolomics with proteomics, transcriptomics, and hiPSC-based platforms strengthens causal inference and enables the development of early warning biomarker panels. When embedded within HFA/ICOS-informed risk stratification and multidisciplinary monitoring pathways, these panels can identify patients who require timely cardioprotective measures. Preclinical data supporting metabolomics-guided interventions, most notably L-carnitine for sunitinib-induced metabolic blockade, illustrate the potential to restore substrate utilization and attenuate inflammation, oxidative stress, and fibrosis.

Moving forward, prospective validation of metabolite panels, standardized multi-omic workflows, and biomarker-driven interventional trials are essential to translate these insights into clinical practice. By aligning mechanistic understanding with actionable assays and targeted prevention, precision cardio-oncology can preserve oncologic efficacy while minimizing cardiovascular harm.

## Figures and Tables

**Table 1:** Selected TKIs: cardiotoxic mechanism and supporting study.

TKI	Primary Proposed Cardiotoxic Mechanism	Supporting Study
Sunitinib	AMPK-α2 inhibition → ↑ACC2 → ↑malonyl-CoA → ↓CPT1 activity; myocardial carnitine depletion; impaired LCFA oxidation; mitochondrial dysfunction; inflammation/fibrosis	Sayed-Ahmed et al. [28], Cardiovasc Toxicol. 2019
Sorafenib	Mitochondrial complex I downregulation; ER stress (PERK–eIF2α–ATF4); ROS generation; taurine depletion; impaired Ca^2+^ handling	Jensen et al. [29], Metabolites. 2017
Imatinib	PGC-1α downregulation → impaired mitochondrial biogenesis; altered fatty-acid oxidation; direct myocyte effects in some models	Li et al. [30], Cardiovasc Innov Appl. 2023
VEGFR-directed TKIs (e.g., axitinib, pazopanib, sorafenib, sunitinib)	VEGF–VEGFR2 blockade → loss PI3K–Akt–eNOS signaling; Nox activation → ROS/ONOO^−^; eNOS uncoupling; endothelial apoptosis and capillary rarefaction → hypertension, ischemia	Touyz et al. [31], 2018; Eremina et al. [32] (renal microangiopathy)
Ibrutinib (BTK inhibitor)	Off-target effects on cardiac ion channels and signaling → atrial arrhythmogenesis; possible direct myocardial effects in susceptible genotypes	Herman SEM et al. [33], Clin Cancer Res. 2017

**Table 2:** Differential metabolites linked to TKI induced cardiotoxicity.

Metabolite	Direction (TKI Exposure)	Sample	Pathway	Study
Branched-chain amino acids (Leu/Ile/Val)	↑	Plasma; cardiac tissue	Mitochondrial substrate overload; ROS generation	Jensen et al. [29], Metabolites 2017; Li et al. [30], CVIA 2023
Aromatic amino acids (Phe, Tyr, Trp)	↑	Plasma	Kynurenine pathway activation; inflammation	Jensen et al. [29], Metabolites 2017; Li et al. [30], CVIA 2023
Creatine	↑	Plasma; cardiac tissue	Impaired oxidative phosphorylation; ATP depletion	Jensen et al. [29], Metabolites 2017; Sayed-Ahmed et al. [28], Cardiovasc Toxicol 2019
Mannitol (osmolyte proxy)	↑	Plasma/urine	Endothelial permeability; TonEBP/NFAT5 signaling	Osmotic-stress literature; Kültz & Burg [71], 1998; López-Rodríguez et al. [72], 2004
Taurine	↓	Cardiac tissue; plasma	Osmoregulation; Ca^2+^ handling; antioxidant buffering	Jensen et al. [29], Metabolites 2017; Duran et al. [68] 2014
Long-chain PUFAs (DHA, EPA, AA)	↓	Cardiac tissue; plasma	Membrane integrity; anti-inflammatory signaling	Jensen et al. [29], Metabolites 2017;
Acyl-carnitines (short/long chain)	↑ or ↓ (chain-dependent)	Cardiac tissue; plasma	LCFA transport/β-oxidation; CPT1 axis	Jensen et al. [29], Metabolites 2017; Sayed-Ahmed et al. [28], Cardiovasc Toxicol 2019
Adenylate metabolites (AMP, ADP, ATP intermediates)	↓	Cardiac tissue	Bioenergetic failure; impaired OXPHOS	Jensen et al. [29], Metabolites 2017
Nitrotyrosine/nitrosative markers	↑	Cardiac tissue; plasma	eNOS uncoupling; oxidative/nitrosative stress	Neves et al. [31], Hypertension 2018; preclinical VEGFR inhibition studies
Malonyl-CoA (via ACC2)	↑	Cardiac tissue	Inhibits CPT1; reduces LCFA mitochondrial uptake	Sayed-Ahmed et al. [28], Cardiovasc Toxicol 2019; sunitinib mechanistic studies

## Data Availability

No new data were created or analyzed in this study.

## Abbreviations

The following abbreviations are used in this manuscript:

AA	Arachidonic Acid
ACC2	Acetyl-CoA Carboxylase 2
ADP	Adenosine Diphosphate
AMP	Adenosine Monophosphate
AMPK	AMP-Activated Protein Kinase
ATP	Adenosine Triphosphate
ATF4	Activating Transcription Factor 4
CaMKII	Calcium/Calmodulin-Dependent Protein Kinase II
CoA	Coenzyme A
CPT-1	Carnitine Palmitoyltransferase 1
CPT-2	Carnitine Palmitoyltransferase 2
CV	Cardiovascular
DHA	Docosahexaenoic Acid
EI	Electron Ionization
eIF2α	Eukaryotic Initiation Factor 2 Alpha
eNOS	Endothelial Nitric Oxide Synthase
EPA	Eicosapentaenoic Acid
ESI	Electrospray Ionization
FDG	Fluorodeoxyglucose
FS	Fractional Shortening
GC–MS	Gas Chromatography–Mass Spectrometry
hOCTN2	Human Organic Cation/Carnitine Transporter 2
IHC	Immunohistochemistry
JAK–STAT	Janus Kinase–Signal Transducer and Activator of Transcription
LC–MS	Liquid Chromatography–Mass Spectrometry
MALDI	Matrix-Assisted Laser Desorption/Ionization
MAPK	Mitogen-Activated Protein Kinase
MS	Mass Spectrometry
NMR	Nuclear Magnetic Resonance
NO	Nitric Oxide
Nox	NADPH Oxidase
ONOO^−^	Peroxynitrite
PDGF	Platelet-Derived Growth Factor
PDGFR	Platelet-Derived Growth Factor Receptor
PERK	Protein Kinase RNA-Like Endoplasmic Reticulum Kinase
PGC-1α	Peroxisome Proliferator-Activated Receptor Gamma Coactivator 1-Alpha
PGI2	Prostacyclin
PI3K	Phosphoinositide 3-Kinase
PUFA(s)	Polyunsaturated Fatty Acid(s)
ROS	Reactive Oxygen Species
TKI(s)	Tyrosine Kinase Inhibitor(s)
TCA cycle	Tricarboxylic Acid Cycle
TXA2	Thromboxane A2
VEGF	Vascular Endothelial Growth Factor
VEGFR	Vascular Endothelial Growth Factor Receptor
